# Pedagogical psychology: beyond the 21st century

**DOI:** 10.3389/fpsyg.2015.00280

**Published:** 2015-03-11

**Authors:** Gretchen M. Reevy, Stanley N. Bursten

**Affiliations:** ^1^Psychology, California State University, East BayHayward, CA, USA; ^2^Psychology, Santa Barbara City CollegeSanta Barbara, CA, USA

**Keywords:** education, teaching, intelligent virtual agents, neuroscience of learning, critical thinking, technology and pedagogy, online and hybrid delivery, faculty issues

The need of a society to educate its young in order to help them become valuable members of that society was likely recognized even before historical records began accumulating. The classic philosophers recognized this need even as they thought about other issues (Tweed and Lehman, 2002).

However, it appears that pedagogical theory has remained relatively stagnant, focusing on the teaching-by-lecture model until well into the 20th century (Landrum, 2009; Berrett, 2014). As governments and citizenry began realizing that older methods of education were not optimally preparing students to become productive and self-fulfilling members of society, societies began examining older approaches to education and new, hopefully more effective methods have been developed.

This special topic in *Frontiers in Educational Psychology* presents papers that exemplify only a few of the emerging theoretical and applied approaches toward pedagogy that aim to produce the competent graduates society expects from its learning institutions. Rather than focusing on pedagogical issues directly relevant to classic classroom content (e.g., basic skills, foundations of chemistry or history), the articles presented here focus on ideas which can form a basis for emerging and future applications. Thus, instead of bringing together articles which share an easily identifiable common theme, the articles in this issue are connected by an overarching goal—presenting new and sometimes untried approaches.

Several papers in this issue discuss the use of virtual agents or virtual reality in teaching. Macedonia, Groher, and Roithmayr show that second language instruction, particularly of vocabulary, can be more efficient through utilizing an intelligent virtual agent (IVA) than through using a human as the instructor. In an opinion piece, Repetto agrees with Macedonia et al. that IVAs are a promising vehicle for teaching second languages, and that the teaching and investigation of second language learning could be further enhanced through utilizing virtual reality. As Repetto describes, using virtual reality means that the learner is engaged in physical movement while learning, which bolsters memory. In a third paper Macedonia reviews research on use of gestures while learning the vocabulary of a second language, presenting evidence of the positive effects of movement while learning language. Although the articles focus on language learning, the principles described by these authors could apply to learning in other areas.

Schmaltz and Lilienfeld discuss an engaging way to teach critical thinking. They suggest presenting pseudoscience claims in class (e.g., paranormal phenomena), and then require that students closely examine these claims. By comparing pseudoscience claims with claims derived from scientific methods, students sharpen their critical thinking skills. Universities or government agencies in many nations now recognize critical thinking as an essential learning outcome for high school or college graduates [e.g., in the United States (Association of American Colleges and Universities, 2005) and Canada (Premier's Technology Council, 2010)]. In a novel approach, Binnun and Tarrasch describe a method for incorporating contemplative exercises, which they call “personal brain investigations” into a neuroscience course. They explain that some students in the humanities and social sciences have difficulty accepting some aspects of neuroscience, for instance, its reductionism; thus, adding an experiential component can enhance understanding and retention. The authors present survey results from students, revealing that many students reported satisfaction with the exercises, agreeing that the exercises produced benefits, such as discovery of new dimensions in relation to brain and mental processes.

Several papers address the growing use of technology to supplement and improve the traditional lecture. Gernsbacher describes the benefits of online courses over face-to-face. For instance, online courses can readily be designed to produce distributed learning, which leads to better mastery of course material. Van Doorn and Van Doorn's review presents a somewhat different perspective. The authors conclude that, in many cases, hybrid (blended) courses (which combine features of face-to-face and online) may be the best platform for many students, particularly non-traditional students. The authors also present a typology of the differing learning styles and needs of traditional and non-traditional students. Gernsbacher's and the Van Doorns' discussions are highly relevant in the 21st century; in 2013, one-third of university students in the United States took at least one online course (Allen and Seaman, 2013). Schmaltz and Enstrom describe how to most effectively use PowerPoint in teaching, explaining that university instructors are rarely taught how to use PowerPoint prior to entering the classroom.

In an opinion piece, Calder Stegemann discusses 21st century goals of teaching as identified by government agencies in Canada (e.g., Premier's Technology Council, 2010), and explains that pedagogical approaches to courses can be modified in order to achieve these new goals. Calder Stegemann specifically describes an approach to teaching educational psychology students. For instance, since a primary goal of educators is to teach students how to learn rather than to teach students information, instructors should devote more time to teaching students how to *acquire* information and less time to the dissemination of information (i.e., lecturing).

The final article, research by Reevy and Deason, is the only article in the issue which does not directly address pedagogy. Instead, the authors discuss an issue which is affecting the ability of higher education institutions to produce the most effective teaching methods: the employment contracts and working conditions of non-tenure-track (NTT) faculty who comprise the majority of faculty in higher education in the United States and other countries. Their study investigated relationships among working conditions, demographic and psychological variables, and measures of well-being in NTT faculty. They found that faculty with lower incomes, higher organizational commitment, and who desired a permanent position experienced elevated levels of depression, stress and anxiety. Since the goal of higher education is to produce the next generations of productive citizens, any variable that negatively impacts pedagogy must be addressed.

A number of societal factors including increasing technology, globalization, and neoliberalism have impacted all primary institutions in societies across the world. These factors have created both challenges to and opportunities for society's goal of educating our citizenry. The ideas and findings presented here in this special issue offer valuable contributions to the growing discussion about how we may best prepare the next generations of global citizens in the 21st century.

## Conflict of interest statement

The authors declare that the research was conducted in the absence of any commercial or financial relationships that could be construed as a potential conflict of interest.
